# Reinforcement Learning Based Artificial Immune Classifier

**DOI:** 10.1155/2013/581846

**Published:** 2013-07-08

**Authors:** Mehmet Karakose

**Affiliations:** Computer Engineering Department, Firat University, Elazig, Turkey

## Abstract

One of the widely used methods for classification that is a decision-making process is artificial immune systems. Artificial immune systems based on natural immunity system can be successfully applied for classification, optimization, recognition, and learning in real-world problems. In this study, a reinforcement learning based artificial immune classifier is proposed as a new approach. This approach uses reinforcement learning to find better antibody with immune operators. The proposed new approach has many contributions according to other methods in the literature such as effectiveness, less memory cell, high accuracy, speed, and data adaptability. The performance of the proposed approach is demonstrated by simulation and experimental results using real data in Matlab and FPGA. Some benchmark data and remote image data are used for experimental results. The comparative results with supervised/unsupervised based artificial immune system, negative selection classifier, and resource limited artificial immune classifier are given to demonstrate the effectiveness of the proposed new method.

## 1. Introduction

**Figure 1:**
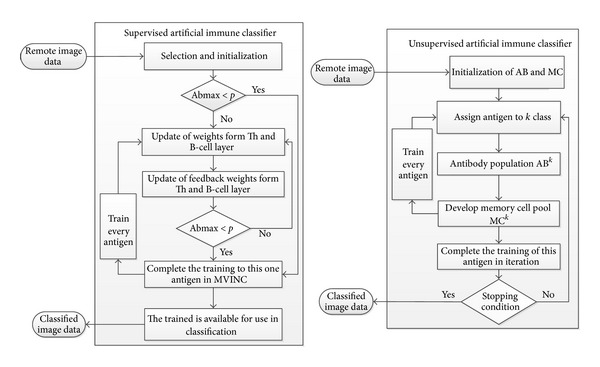
Artificial immune classifiers with supervised-unsupervised learning models [2, 3].

Artificial immune systems are one of the algorithms that forms the basis of classification methods and they can be used in many areas of daily life. The basis of these algorithms is based on the human immune system, and they have been usually used in popular areas such as the classification, anomaly detection, and the computer virus detection. Human immune system known as the natural immune system is an effective mechanism for protecting the human body against foreign cells. By utilizing these features of natural immune systems, artificial immune systems have been developed for solving the scientific and engineering problems. Artificial immune systems are used in many applications such as optimization, classification, and pattern recognition. In classification algorithms, there are various algorithms that are different from artificial immune systems. These algorithms are based on maximum likelihood and minimum distance. They are used on parallel and piped images [1]. These type algorithms used in the classification of images generally need the preclassified data. In classification methods, there are two basic learning method such as supervised and unsupervised learning algorithms. Block diagrams of some artificial immune classifiers in the literature are given in Figure 1.

Supervised and unsupervised learning algorithms are widely used methods in classification problems [2–13]. Some algorithms such as *K*-Means [4], ISODATA, Fuzzy *C*-Means, and AIS (Artificial Immune System) [2] are known as unsupervised classification methods, and they cluster data without the need for the training data. Zhong et al. [2] proposed unsupervised artificial immune classification method for multi-hyperspectral remote sensing images. Four different structures such as water, plants, roads, and buildings are classified by using unsupervised artificial immune system. Afterwards, the obtained results with the results of other methods such as *K*-Means, ISODATA, Fuzzy *C*-Means, and SOM methods were compared. In another study, Zhong et al. [3] proposed supervised artificial immune classification for remote sensing images. The effectiveness of the proposed algorithm is verified via simulation results. Another study of Zhong and Zhang [5] supervised adaptive artificial immune network was proposed for multi/hyperspectral remote sensing images. Aydin et al. [7] proposed an adaptive artificial immune algorithm for fault diagnosis of induction motors faults. The classification was made by using three-phase current signals taken from a real induction motor. Stator and broken rotor bar faults are detected by using this classification method. 

Supervised and unsupervised learning methods have some advantages over one another, though they have some disadvantages according to reinforcement learning. Reinforcement learning presents a wide solution framework in planning and control. This learning method aims to obtain most suitable results with award values for each action of an agent. The purpose of the training process continues until agent reaches the awards. Q learning algorithm is a reinforcement learning algorithm. Q learning algorithm is based on status and action value of Q and converges according to award value of action and Q values of next status. The reinforcement learning has been used for many applications such as fuzzy systems, neural networks, and classification applications [8–11].

This paper presents a new artificial immune classifier based on reinforcement learning. The proposed approach has a self-learning structure using clonal selection and memory cells. Especially this algorithm is trained by reinforcement learning with feedback of mutation rate. Thus best antibodies are obtained to recognize antigens. In this paper, Section 2 presents algorithms for artificial immune systems.  Section 3 provides details of the proposed new approach.  Section 4 presents the validation of the proposed approach using experimental results and Section 5 gives the conclusions.

## 2. Artificial Immune Systems

Artificial immune system was exposed by inspiring the human immune system. The method is used in many fields, especially engineering [12]. Artificial immune systems have been developed to detect and destroy viruses with the emergence of computer viruses and used in the antivirus detection process. Immune systems are effective protection mechanisms that protect the human body from foreign antigens or pathogens [13]. The artificial immune algorithm has been uncovered by considering the importance of the artificial immune system in human life. The base of the artificial immune algorithm is the same with artificial immune system. Furthermore, the method has become indispensable for optimization and classification problems. Different types of methods have emerged with creation of the artificial immune algorithm. Some of these methods have become important algorithms which form the basis of the artificial immune algorithm. Artificial immune systems basically consist of two sections. These important algorithms are clonal selection algorithm and negative selection algorithm.

*The clonal selection algorithm* is based on the cloning of immune cells in type B antigen recognition. The structures that will be used as a set of data structures for the algorithm have been applied in the clonal selection structure. The pseudocode of the clonal selection algorithm is given in Pseudocode 1. The algorithm that used in pattern recognition is shown in Pseudocode 1. The similarities between antibodies and antigens are found and the clones are formed from antibodies with high similarity.

*The negative selection algorithm* is often used in determination of the undesirable situations. Particularly, this method is use in the operating of virus detection software. The implementation and the operation of the algorithm are quite easy. First, a set of is produced with this method and this candidate detector set is matched with the self-set. If there is a matching, these candidates are transferred to the main detector set. Afterwards, the detector set compared by entering the test data. If this test data is recognized by any detector, the desired operation is performed. The training and the test phase of the negative selection algorithm are expressed with pseudocode given in Pseudocode 2.

## 3. The Proposed Approach

**Figure 2:**
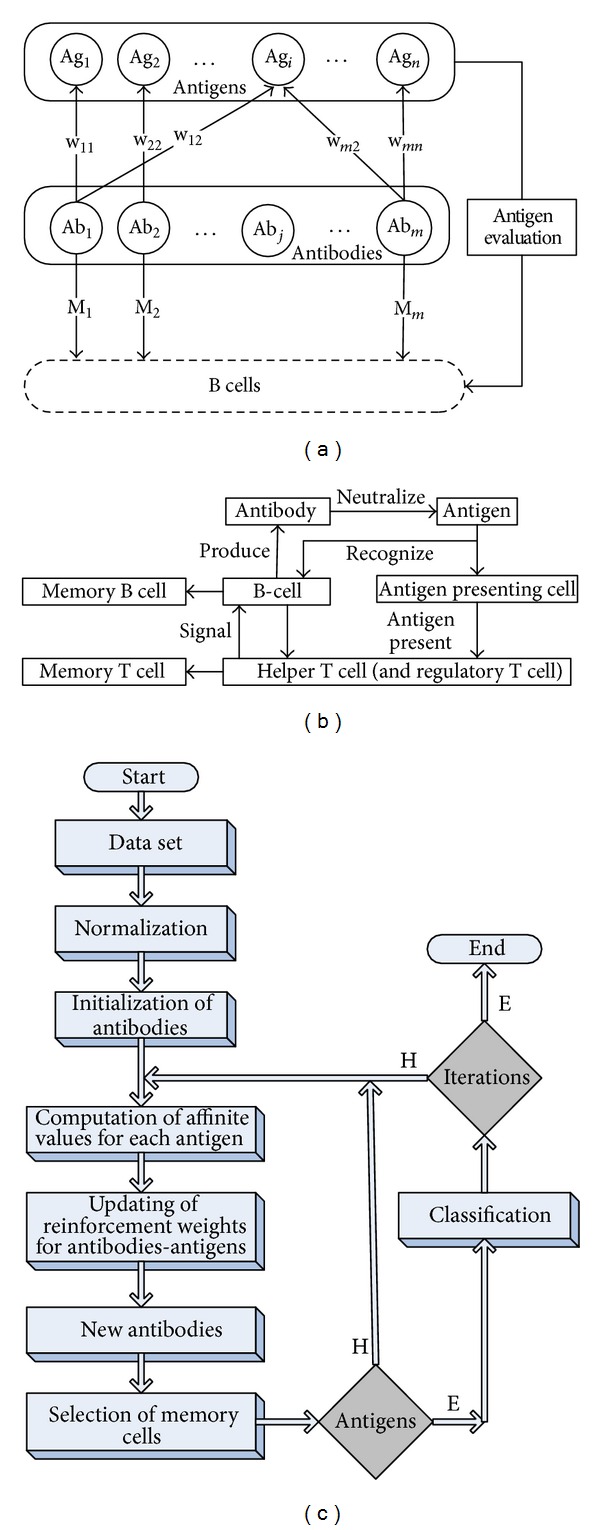
Block diagram and flow chart of the proposed approach.

The reinforcement based artificial immune classifier is proposed for classification in this study. Reinforced artificial immune classifier method can be easily used in all areas in which using the computational intelligence is convenient and can produce the best results in used areas. This method is welded from reinforcement learning algorithm which is a machine learning method. Successful results were obtained by using reinforcement learning algorithm in machine learning. The difference of reinforcement learning algorithm from other algorithms is to calculate all probabilities of current states to identify the next state. So, each step that algorithm operates is used as reinforcement for the next operation. The reinforced artificial immune classifier is composed by using this algorithm in combination with an artificial immune system. The flow chart of proposed method is given in Figure 2.

Q learning algorithm is a reinforcement learning algorithm. According to the Q learning algorithm, the existing situation and the Q value of its action converge with a certain coefficient to the award value of the action and Q values of the arriving situations. In the Q learning algorithm, the situation of the algorithm for step and the best possible step for next situations are determined by considering this situation. The Q value of the next situation in algorithm is calculated by (1)

(1) Q(status,action)  =R(status,action)   +σmax⁡(Q(next  status,all  mutation  actions)).

The *R* value given in (1) indicates the current status of the algorithm. *σ*max⁡ value is the most high affinity value of the steps for the next situation. The pseudocode of Q learning algorithm is given in Pseudocode 3.

Q learning is a commonly used algorithm in reinforcement learning methods. A new reinforced artificial immune classifier was proposed by applying the algorithm given the steps in Figure 2 to artificial immune system. Each antigen is recognized by an antibody (Ab). The connection between antigens and antibodies is updated according to Q learning algorithm in reinforced learning. Antibodies turn to the antigens is achieved through the clonal selection and mutation operators in immune selection. Antibodies that recognize the antigens are formed B-cell cells as memory cell. The pseudo code of proposed algorithm is given in Pseudocode 4.

In this study, the set of antigens have been identified as training data. Each antigen is represented by Ag, and the set of each antigen is expressed by AG. The set of antibodes is started in solution space at random locations. Each antibody is represented by Ab, and the set of antibodes is expressed by AB. The affinity of any *i*. Antibody is calculated by two factors given in (2) and (3):

(2) $$\begin{array}{c} D_{s}=\sum_{j\in \{d\}}\frac{1}{d_{ij}+1}, \end{array}$$

(3) $$\begin{array}{c} Y_{s}=\sum_{j\in \{y\}}\frac{1}{d_{ij}+1}. \end{array}$$

The set of *d* indicates the number of antigens that are correctly classified by *i* antibody in the previous equations. The set of misclassified samples with the same antibody is expressed with *y* in the second equation. According to these two equations, the affinity account is maintained as (4)

(4) $$\begin{array}{c} \text{ab}\cdot \text{af}=\{\begin{array}{cc} \frac{D_{s}}{N}+2.0, & \text{If}\left\{d\right\}\neq 0\;\text{ve}\left\{y\right\}=0,\\ \frac{D_{s}-Y_{s}}{D_{s}+Y_{s}}+1.0, & \text{If}\left\{d\right\}\neq 0\;\text{ve}\left\{y\right\}\neq 0,\\ 0, & \text{If}\left\{d\right\}=0\;\text{ve}\left\{y\right\}=0. \end{array} \end{array}$$

Each antigen is recognized by an antibody. Each connection of an antibody with antigens is updated as (5)

(5) $$\begin{array}{c} w_{ij}\left(t+1\right)=w_{ij}\left(t\right)+\alpha \left(r_{ij}\left(t+1\right)-w_{ij}\left(t\right)\right). \end{array}$$

In (5), *α* indicates learning constant and *r*_*ij*_(*t*) indicates the award obtained in step *t*. The affinity value is used as an award here. Learning constant *α* is determined in [0,1]. If an antibody recognizes an antigen, the antibody memory cell turns into (B cell). The weight value between the antibody turned into the cell and B cell, *M*_*k*_ is 1. This value determines the excitation level of related B cell. The antibodies transformed memory cell before is updated according to (6)

(6) $$\begin{array}{c} M_{k}=\beta M_{k}. \end{array}$$

*β* is the reduction factor used in (6) and gets value in [0,1]. The clones of antibodies are generated proportional with their affinity. The clone are not created for antibodies whose affinities are over 2.0. The clones are formed as (7) for antibodies whose affinities are under 2.0

(7) $$\begin{array}{c} N_{c}=\sum_{k=1}^{n}\text{round}\left(c_{\text{rate}}*\text{a}{\text{b}}_{k}\cdot \text{ aff }\right). \end{array}$$

The mutation is applied on the clones obtained in (7) according to affinity values. The mutation process is maintained as claimed in (8)

(8) Abc(i)=Abc(i)+rand(−s,s),if  d<mutaterate.

(*d*) number is a random number generated in [0,1]. If this number is lower than mutation possibility, the mutation is applied to this antibody gene. The best mutated clone of an antibody is selected as a memory cell. According to the obtained memory cells, the performed classification process depending on the nearest neighbor algorithm is given in (9):

(9) Classification  performance  =Successful  classification  samplesTotal  samples∗100.

## 4. Experimental Results

The proposed reinforcement learning based artificial immune classifier has been implemented using software written in Matlab m-file and hardware in FPGA. The Iris data and remote image data were used to evaluate performances of the proposed approach and other artificial immune classifiers. 

**Figure 3:**
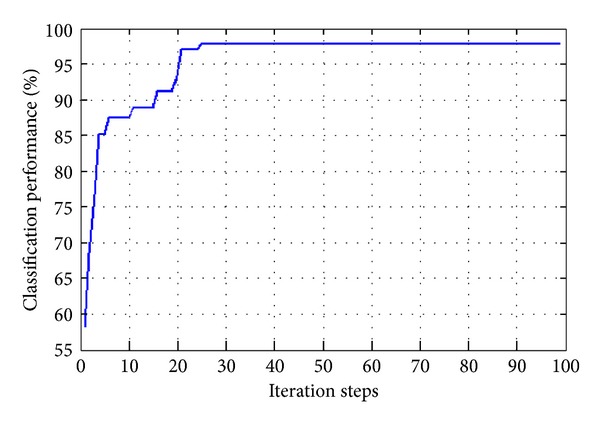
A test result for iris data.

Iteration  steps: firstly, performance of the proposed approach has been tested using the iris data. The iris controled the diameter and size of the pupil is a thin and circular structure in the eye. 768 × 4 matrix obtained by processing of iris images is used for classification and results is compared other artificial immune classification methods. In this process, data is divided into 10 parts, 9 parts are used for training, and one part is also used for testing of algorithm. Performance of algorithm is obtained by average of results computed with 10 test processes. The classification performance of the proposed approach for iris data is shown in Figure 3. The comparative results between the proposed approach and other artificial immune classifiers have been given in Table 1. Conventional artificial immune classifiers have large classification time for large data size. In addition, they use many memory cells for classification process. As shown in Table 1, the proposed approach has more effective classification performance and less memory cells according to other methods.

**Figure 4:**
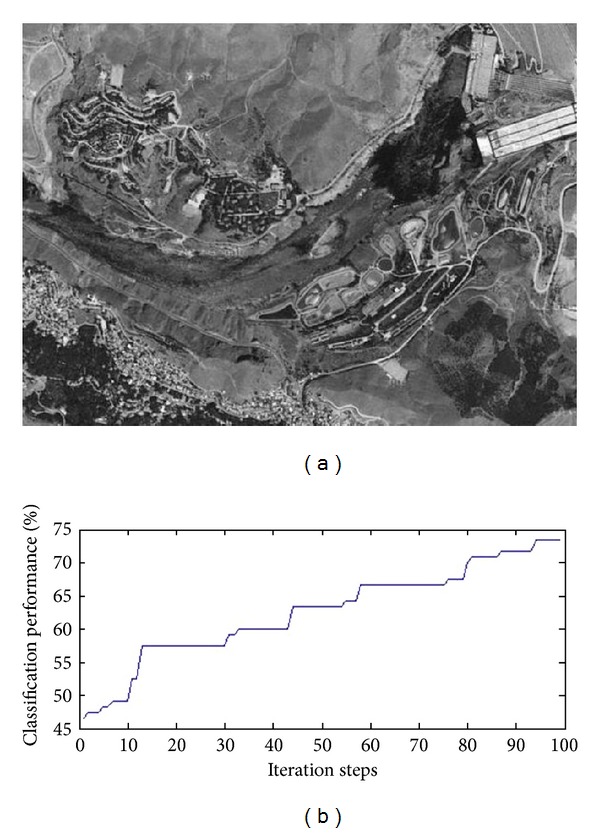
(a) Example remote image (400 × 400 pixel). (b) Performance graphic.

Secondly, performance of the proposed approach has been tested using the remote image data. 400 × 400 pixel color image has been used for this purpose. If image has plant, water, building, and ways areas, performance and effectiveness of the proposed approach can be well presented.  Figure 4 and Table 2 show an image used for experiments and comparative classification performance of the proposed approach.

## 5. Conclusions

Artificial immune systems have an important role for classification methods that are commonly used in real-world applications. However, there are many approaches to improve the performance of artificial immune systems in the literature. For this purpose, supervised and unsupervised learning algorithms are used in many studies. But artificial immune systems that used supervised or unsupervised learning algorithms have many disadvantages in terms of today's data features. Therefore, a new artificial immune classifier based on reinforcement learning has been proposed for classification applications in this study. The proposed approach that used reinforcement learning algorithm for computation of memory cells of artificial immune system aimed to obtain a new classifier that is faster in real time, has less memory cells, has higher accuracy in results, and more effectively. Performance and effectiveness of the proposed approach has been shown using some benchmark data and remote image data. Comparative results between proposed approach and some methods in the literature have been given with experimental results. The reinforcement learning based artificial immune classifier provides higher accuracy with less memory cells as seen in the experimental results.

## Figures and Tables

**Pseudocode 1:**
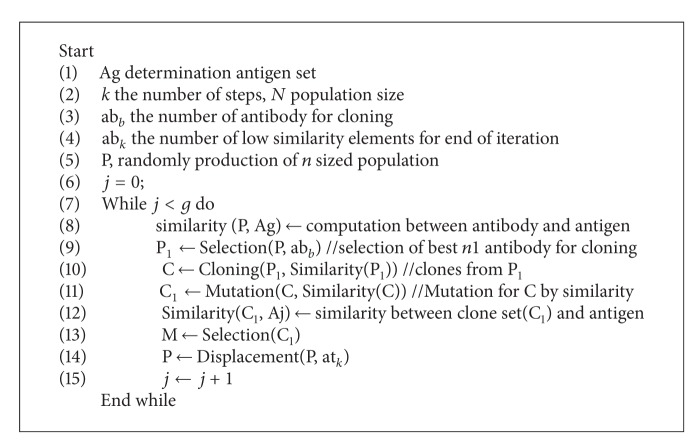
The pseudocode of clonal selection algorithm.

**Pseudocode 2:**
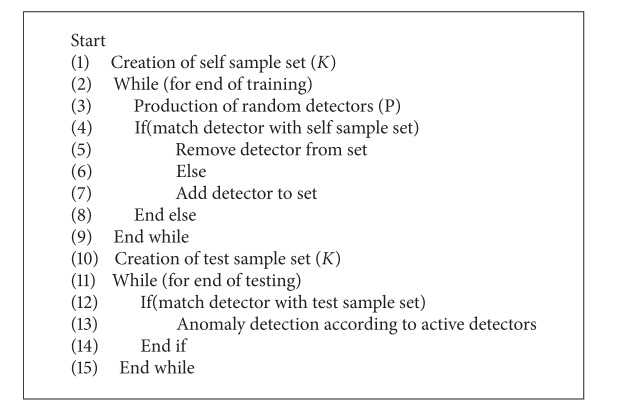
The pseudocode of negative selection algorithm.

**Pseudocode 3:**
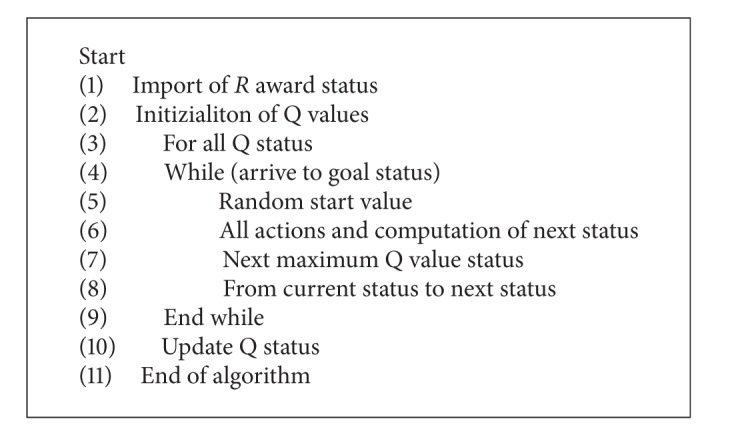
The pseudocode of Q learning algorithm.

**Pseudocode 4:**
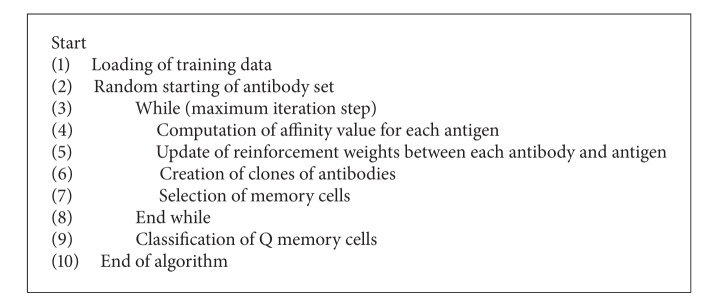
The pseudocode of reinforcement learning based artificial immune classifier.

**Table 1:** Comparative results for the proposed approach.

Classification methods	Iris	Wine	Sonar
	Classification performances		
Proposed classifier	**97.33 ± 3.06**	**97.6 ± 3.98**	**92.17 **±** 2.92**
[5]	96.3 ± 4.7	96.1 ± 4.7	—
[3]	94.8	—	89.1
[2]	95.7	—	84.9

	Number of memory cells		
Proposed classifier	**21**	**30**	**60**
[5]	100	100	—
[3]	28	—	71
[2]	32	—	177

**Table 2:** Comparison of results for the proposed approach.

Class	Samples	Award function values	[5]	[1]	Proposed approach
Water	950	00111100,0010111,00011000	97%	96%	**99%**
Plant	350	00110000,00111000,010110	82%	78%	**84%**
Road	250	00000011,00010110,00000110	85%	—	**88%**
Building	300	10100110,0110011,0101111	80%	82%	**85%**
